# Dopamine D2 −141C Ins/Del and Taq1A polymorphisms, body mass index, and prediction error brain response

**DOI:** 10.1038/s41398-018-0147-1

**Published:** 2018-05-23

**Authors:** Guido K. W. Frank, Megan E. Shott, Marisa C. DeGuzman, Andrew Smolen

**Affiliations:** 1 Department of Psychiatry, University of Colorado School of Medicine, University of Colorado Anschutz Medical Campus, Aurora, CO USA; 2 Neuroscience Program, University of Colorado Denver, Anschutz Medical Campus, Aurora, CO USA; 3 University of Colorado Boulder, Institute for Behavioral Genetics, Boulder, CO USA

## Abstract

The prediction error model is a widely used paradigm that is conceptually based on neuronal dopamine function. However, whether dopamine receptor gene alleles contribute to human neuroimaging prediction error results is uncertain. Recent research implicated the dopamine D2 receptor in behavior response during a prediction error paradigm and we expected that polymorphisms of that receptor would contribute to prediction error brain response. In this study, healthy female participants in the early follicular phase of the menstrual cycle underwent a taste prediction error paradigm during functional magnetic resonance imaging. Participants were also genotyped for dopamine receptor polymorphisms. Our data suggest that the dopamine D2 receptor −141C Ins/Del and Taq1A polymorphisms together with body mass index selectively explain putamen prediction error response. This was true using a region of interest analysis as well as for a whole-brain analysis (FWE corrected). Polymorphisms for dopamine D1 or D4 receptors, dopamine transporter, or COMT did not significantly contribute to prediction error activation. The prediction error model is a computational reward-learning paradigm that is important in psychiatric research and has been associated with dopamine. The results from this study indicate that dopamine D2 receptor polymorphisms together with body mass index are important determinants to include in research that tests prediction error response of the brain. Psychiatric disorders are frequently associated with elevated or reduced body weight. Adding BMI to genetic information in brain-imaging studies that use reward and the prediction error paradigm may be important to increase validity and reliability of results.

## Introduction

Brain reward response has been associated with dopamine (DA) function^1^. However, identifying how DA genotype contributes to this brain activation has been challenging^2^. Previous studies used a “multilocus” composite DA genotype approach, where an additive score was calculated based on a presumed DA signal-enhancing vs. -reducing alleles^2,3^. This strategy requires knowledge of the biochemical significance of each allele on DA signaling, though, and it has been uncertain if the biological effects of the polymorphisms are additive^2^.

Those referenced previous studies had applied brain-imaging tasks for receipt or anticipated receipt of taste or monetary reward stimuli, or a game that involved guessing numbers to win money^2,3^. However, reward processing involves a complex brain circuitry and various neurotransmitters, and those tasks were not necessarily anchored in a model for DA-related brain response^4^. An approach that has been associated with neuronal DA response is reinforcement learning, especially in the context of unexpected receipt or omission of reward stimuli^1^. Midbrain dopaminergic neurons exhibit a phasic burst when receiving unexpected reward (“positive prediction error”), and will shift the signal to the onset of a conditioned stimulus, which they have learned will predict reward receipt^1^. A negative prediction error (PE) (dip in DA neuron activity) is evoked when the predicted stimulus association is violated (unexpected reward omission, negative PE). In this model, the so-called temporal difference algorithm, a PE can be calculated based on expectation and reward outcome, which directly relates to changes in phasic or tonic DA neuron activity^1,5,6^. Human brain-imaging studies have shown that especially the ventral striatum (caudate, putamen) and midbrain are responsive to or code the PE^6^.

The PE model is part of the National Institute of Mental Health (NIMH) research domain criteria initiative (RDoC, positive valence, approach motivation) and has therefore become highly relevant for psychiatric research^7,8^. Understanding the involvement of specific neurotransmitter receptors in this model is of critical importance to identify specific pharmacological targets when studying disorders associated with altered DA and reward function. Although DA-D1 and DA-D2 receptors have been associated with PE response, the specific receptor alleles involved have been elusive, and this distinction of function has also been questioned^9,10^. Specifically, those reports hypothesize that phasic DA signals encode unexpected rewards and are associated with behavioral activation through D1 receptors that have low DA affinity. On the other hand, avoidance learning and behavior inhibition are mediated through the high DA affinity D2 receptors. However, such a dichotomous view may not be correct, and those receptor systems may be working more in concert than antagonistically^9,10^. Recent research has implicated in particular the DA-D2 receptor (DA-D2R) and one of its polymorphisms, the Taq1A allele, in a behavioral PE reinforcement learning task^11^. However, we are not aware of studies that have investigated the effects of DA gene polymorphisms on human PE brain response.

In this study, we investigated the role of DA-D2R alleles in driving PE response in the human brain using functional magnetic resonance brain imaging (fMRI). Eisenegger et al.^11^ indicated that the DA-D2R would be involved in PE response. But the direction in which allele frequency would drive brain activation has been elusive. We therefore decided to use linear regression modeling to test those effects. Previous data comparing individuals with anorexia nervosa and obesity suggested that body mass index (BMI) could also contribute to PE response^12^. Starvation is associated with certain adaptations, which drive food intake^13–15^, including changes in DA release and receptor expression^15^. Similarly, overeating and associated weight gain cause changes in the DA system, and specifically in DA-D2R expression^16^. Those described changes occur on a continuum between under-, normal- and overweight individuals^12^, and we therefore hypothesized that BMI would explain at least part of the variance of this response in healthy controls in addition to DA-D2 genotype. For completeness, we also explored whether we would find specific contribution of DA-D1 receptor (DA-D1R) alleles to PE response, as well as polymorphisms for the catechol-o-methyltransferase (COMT), DA transporter (DAT), or the DA D4 receptor (DA-D4R), which had been included in previous studies^2,3^.

## Subjects and methods

### Study participants

Thirty-three healthy Caucasian females were included in the study, which was approved by the Colorado Multiple Institutional Review Board. All study participants signed informed consent. Participants ranged in age from 16 to 43 years (mean 24.3 ± 7.3) and were of normal BMI, ranging from 18.1 to 24.2 (M 21.6 ± 1.4), based on the Centers for Disease and Prevention definition. Study subjects did not have a history of major medical illness and were free from DSM-5 psychiatric diagnoses as determined by a structured clinical interview conducted by a doctoral level interviewer^17^. All women were studied during the early phase of the follicular cycle^18^. This study included novel analyses and we did not have prior data to base the adequate sample size on. However, previous brain-imaging studies with 20–30 individuals per group provided significant correlational results and we expected that over 30 subjects per group would be adequate^19^.

### Genotyping

Participants were asked to provide saliva, from which epithelial cells were collected, using a commercial product, Oragene^®^ (DNAgenotek, Kanata, Ontario, Canada). DNA was extracted from the samples using standard salting-out and solvent precipitation methods, yielding an average of 45 μg of DNA. Methods for genotyping the DAT and DRD4 polymorphisms are detailed by Haberstick et al.^20^

Genotyping of the DA-D2R (ANKK1) Taq1A (rs1800497) and COMT Val158Met (rs4680) SNPs using Taqman^®^ are detailed in Haberstick and Smolen^21^.

The Taqman^®^ assay for the DRD2 −141C Ins/Del (rs1799732) utilized primers and probes from Gemignani et al.:^22^ forward primer: 5′- AAACAAGGGATGGCGGAATC-3′; reverse primer: 5′-CACCAAAGGAGCTGTACCTC-3′; del probe: 5′-VICCAACCCCTCCTACCCGTTCAGGC-MGB-3′; Ins probe: 5′-FAM-CCCTCCTACCCGTTCCAGGCMGB-3′. Taqman^®^ assays for three SNPs in DRD1, rs686 (C___1011786_10), rs4532 (C___1011777_10), and rs5326 (C__11157157_10) were purchased from Thermo Fisher Scientific. All SNP assays were performed according to manufacturer’s protocols using TaqMan^®^ Genotyping Master Mix in an ABI 7000 real-time PCR system in a total volume of 15 µl containing 2 µl of genomic DNA (≤20 ng).

### Brain-imaging procedures

On the study day, participants arrived between 7.00 and 8.00 AM after an overnight fast, and received a standardized breakfast with the instruction to eat until comfortably full. fMRI was performed between 8.00 and 9.00 AM. Brain images were acquired on a GE Sigma 3T scanner. T2* weighted echo-planar imaging for blood-oxygen dependent (BOLD) functional activity was performed, voxel size 3.4 × 3.4 × 2.6 mm, TR 2100 ms, TE 30 ms, angle 70°, 30 slices, interleaved acquisition, and 2.6 mm slice thickness with 1.4 mm gap. We also acquired structural images (T1, SPGR field of view 22 cm, flip angle 10°, slice thickness 1.2 mm, scan matrix 256 × 256, TR 10, TE 3, voxel size 1.2 mm^3^) for analysis of brain anatomy.

### Classical conditioning task

We adapted the design used by O’Doherty et al.^5^ Individuals received three taste stimuli as unconditioned stimulus (US) during fMRI imaging: 1 M Sucrose solution (100 trials), No solution (100 trials), or artificial saliva (80 trials). Individuals learned to associate each taste stimulus with a unique paired visual conditioned stimulus (CS), a geometric shape, which was only probabilistically associated with its corresponding US: the CS shape for No solution was followed in 20% of the trials by sucrose (unexpected sucrose receipt, positive PE condition), and the CS shape for Sucrose was followed in 20% of trials by No solution (unexpected sucrose omission, negative PE condition). Each visual cue (CS) was presented for 2 s. With the disappearance of the visual cue, simultaneously the taste stimulus (US) was delivered, and a black fixation cross appeared on white background. The taste fluid delivery occurred over 1 s. Inter-trial interval was fixed at 6 s. Subjects were instructed to swish their tongue once, look at the fixation cross, and await the next trial. For each subject, the first 10 trials were fixed CS shape for sucrose followed by the delivery of US sucrose to establish an initial stable association between the CS sucrose shape and US sucrose taste^5^. All other trials were fully randomized without predetermined order. The taste stimuli were applied using a customized programmable syringe pump (J-Kem Scientific, St. Louis, MO) controlled by E-Prime Software (Psychological Software Tools, Pittsburgh, PA), and individual taste applications were triggered by the MRI’s scanner’s radiofrequency pulse^23,24^. Task duration was 28 min.

### Brain-imaging analysis

Brain-imaging data were preprocessed and analyzed using SPM12 software (http://www.fil.ion.ucl.ac.uk/spm/software/spm12/). Data from each subject were realigned to the first volume, normalized to the Montreal Neurological Institute template, and smoothed with a 6-mm FWHM Gaussian kernel. Each image sequence was manually inspected, and subjects with artifacts or movement >3 voxel size were excluded from the analysis. Data were preprocessed with slice time correction and motion parameters were applied as regressors in the first-level analysis.

We extracted mean parameter estimates across all voxels within predefined anatomical regions of interest (http://marsbar.sourceforge.net/) to avoid problems from small volume corrected peak voxel statistics or violation of normal distribution. We explored standard a priori bilateral^25^ reward circuitry regions of interest (Automated Anatomical Labeling Atlas^26^): bilateral caudate head, putamen, substantia nigra, and nucleus accumbens.

### Computational model analysis

To test temporal difference model-related brain response, we modeled each participant’s individual PE signal based on trial sequence^5,27^. The predicted value ($\hat V$) at any time (*t*) within a trial is calculated as a linear product of weights (*w*_*i*_) and the presence of the CS stimulus at time *t*, coded in a stimulus representation vector *x*_*i*_*(t)* where each stimulus *x*_*i*_ is represented separately at each moment in time^5^:

$$\hat V(t) = \mathop {\sum}\nolimits_i {w_ix_i(t).}$$

The predicted stimulus value at each time point *t* in the trial is updated by comparing the predicted value at time *t* + 1 to that actually observed at time *t*, leading to the PE *δ*(*t*):

$$\delta (t) = r(t) + \gamma \hat V(t + 1) - \hat V(t),$$

where *r(t)* is the reward at time *t*. The parameter *γ* is a discount factor, which determines the extent to which rewards arriving sooner are more important than rewards that arrive later during the task, with *γ* = 0.99^5^. The weights *w*_*i*_ relate to how likely a particular US follows the associated CS and are updated on each trial according to the correlation between PE and the stimulus representation:

$${\it{\Delta }}w_i = \alpha \mathop {\sum}\limits_t {x_i(t)\delta (t)}.$$

where *α* is a learning rate. Among various learning rates (0.2, 0.5, 0.7), a slow *α* = 0.7 was the best fit for study groups^5^. The initial reward values were 1 for Sucrose and 0 for No solution. The PE calculated for each trial was modeled as an absolute (reflecting response strength) without separating positive or negative PE trials. This trial-to-trial calculated PE was then regressed with the parameter estimates derived from brain activation across all trials within each subject. Parameter estimates were then extracted for further analysis.

### Statistical analysis

All analyses were conducted using the statistics package SPSS 24 (IBM, Inc.).

We created a composite score for each participant based on each DA receptor's genotype and alleles per participant. For each region of interest, the mean value for the PE model regression parameter estimates was extracted and included in a multiple linear regression analysis. Each region of interest-extracted brain parameter estimate was considered the dependent variable. BMI as well as the DA receptor genotypes were included as independent variables. The primary hypothesis was based on DA-D2R polymorphisms driving PE response together with BMI and those variables were grouped together (PE (dependent)—BMI, DA-D2R polymorphisms (independents)). In addition, the DA-D1R polymorphisms rs686, rs4532, and rs5326 were grouped with BMI (PE (dependent)—BMI, DA-D1R polymorphisms (independents)), as were COMT, DAT, and DA-D4R (PE (dependent—BMI, COMT, DAT, DA-D4R (independents)). Linear regression was performed using the “Enter” method. This method was selected because we did not have a specific hypothesis, which of the independent variables would produce the best prediction equation. This is also the most stringent method for multiple linear regression. In addition, we performed bootstrapping. This resampling method was used to reduce bias. In the multiple regression analysis, we assessed collinearity, and a variance inflation factor of >5 was considered indicating significant collinearity. Non-normally distributed data were rank-transformed before statistical analysis.

We further computed a summary score (addition) for the DA-D2R Taq1A alleles, −141C Ins/Del alleles and BMI and performed a whole-brain regression with PE regression maps. For the DA-D2R 141, the Del/Del genotype was assigned a value of 1, the Ins/Del 2, and Ins/Ins 3; for DA-D2R Taq1A, a value of 1 was assigned to the A1/A1, a 2 to the A1/A2, and a 3 to the A2/A2 genotype. Those values were added to the raw BMI value and regressed with brain response. Regression maps were thresholded at *p* < 0.05 peak voxel FWE corrected. In order to test whether a regression would be region-specific, we created a regression map at *p* < 0.001 uncorrected.

## Results

### Genotype data were within the Hardy–Weinberg equilibrium

**Fig. 1:**
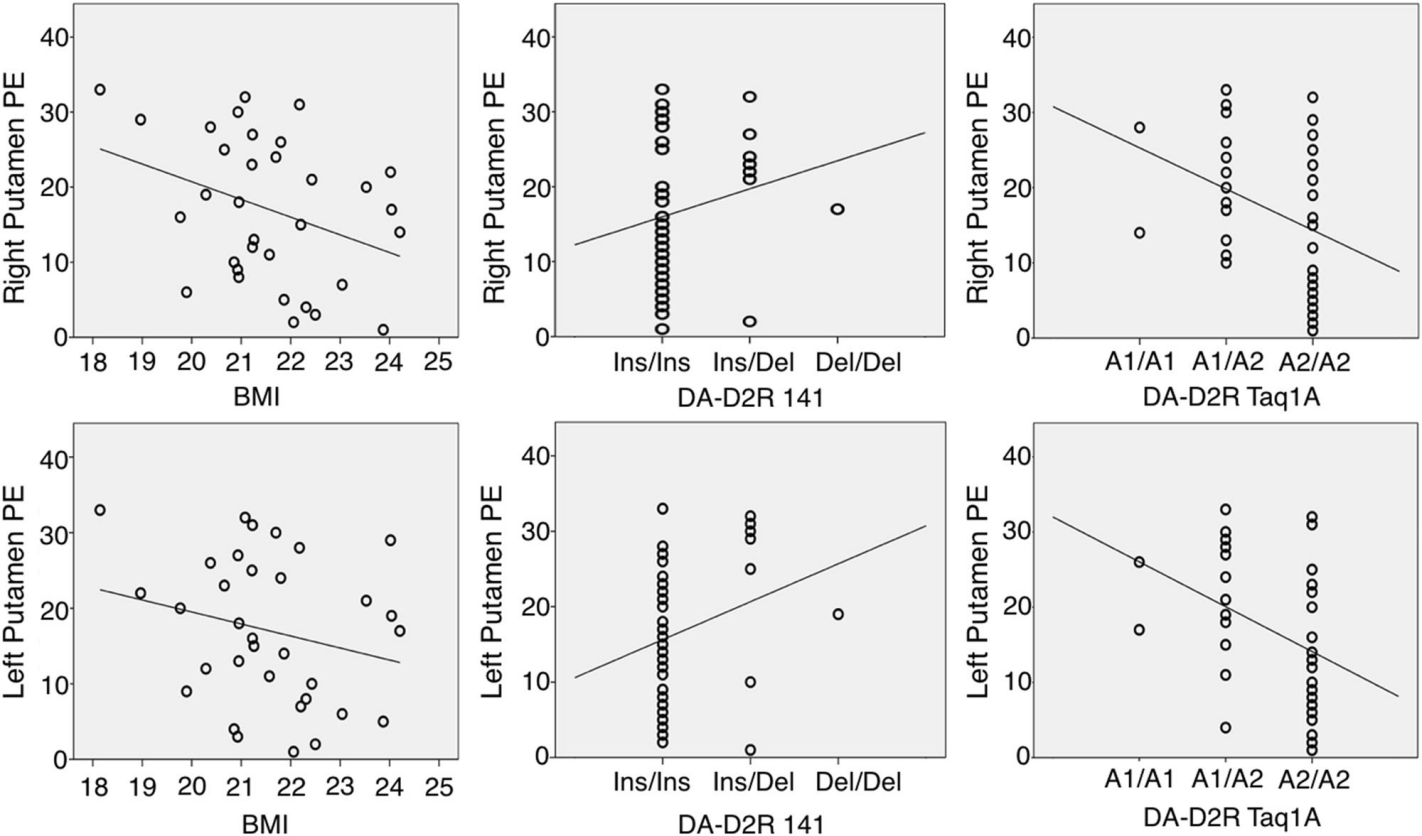
Prediction error correlation plots. Individual scatter plots for body mass index (BMI), DA-D2R alleles for the 141 and Taq1A genotype and bilateral putamen prediction error (PE) values (top panel: right putamen; bottom panel: left putamen)

The combination of DA-D2R Taq1A, −141C Ins/Del and BMI was highly predictive of bilateral putamen PE response and survived multiple comparison correction (Bonferroni), but not for other regions (Table 1, Fig. 1). Figure 1 also shows directionality of DA-D2R allele as well as BMI effects on PE. There was no interaction between BMI and allele frequency. DA-D1R alleles or DAT, COMT, or D4 together with BMI did not significantly predict PE response. However, coefficients for DA-D1R alleles rs686, rs4532 in bilateral nucleus accumbens were significant, and after exploratory removal of the rs5326 allele and BMI, the rs686 and rs4532 alleles predicted left nucleus accumbens PE response (*F* = 3.933, *p* < 0.03), but did not survive multiple comparison correction.

**Table 1:** Multilinear regression results for bilateral putamen; *β*-values are standardized and associated *p*-values derived after bootstrapping

	*β*	*p*	Collinearity tolerance	ANOVA
*Right putamen*				
BMI	−0.556	0.001	0.861	*F* = 7.407, *p* < 0.001
DRD2 −141 Ins/Del	0.396	0.018	0.887	
DRD2 Taq1A	−0.461	0.001	0.96	
*Left putamen*				
BMI	−0.457	0.002	0.861	*F* = 6.592, *p* < 0.002
DRD2 −141 Ins/Del	0.434	0.035	0.887	
DRD2 Taq1A	−0.477	0.001	0.96

**Fig. 2:**
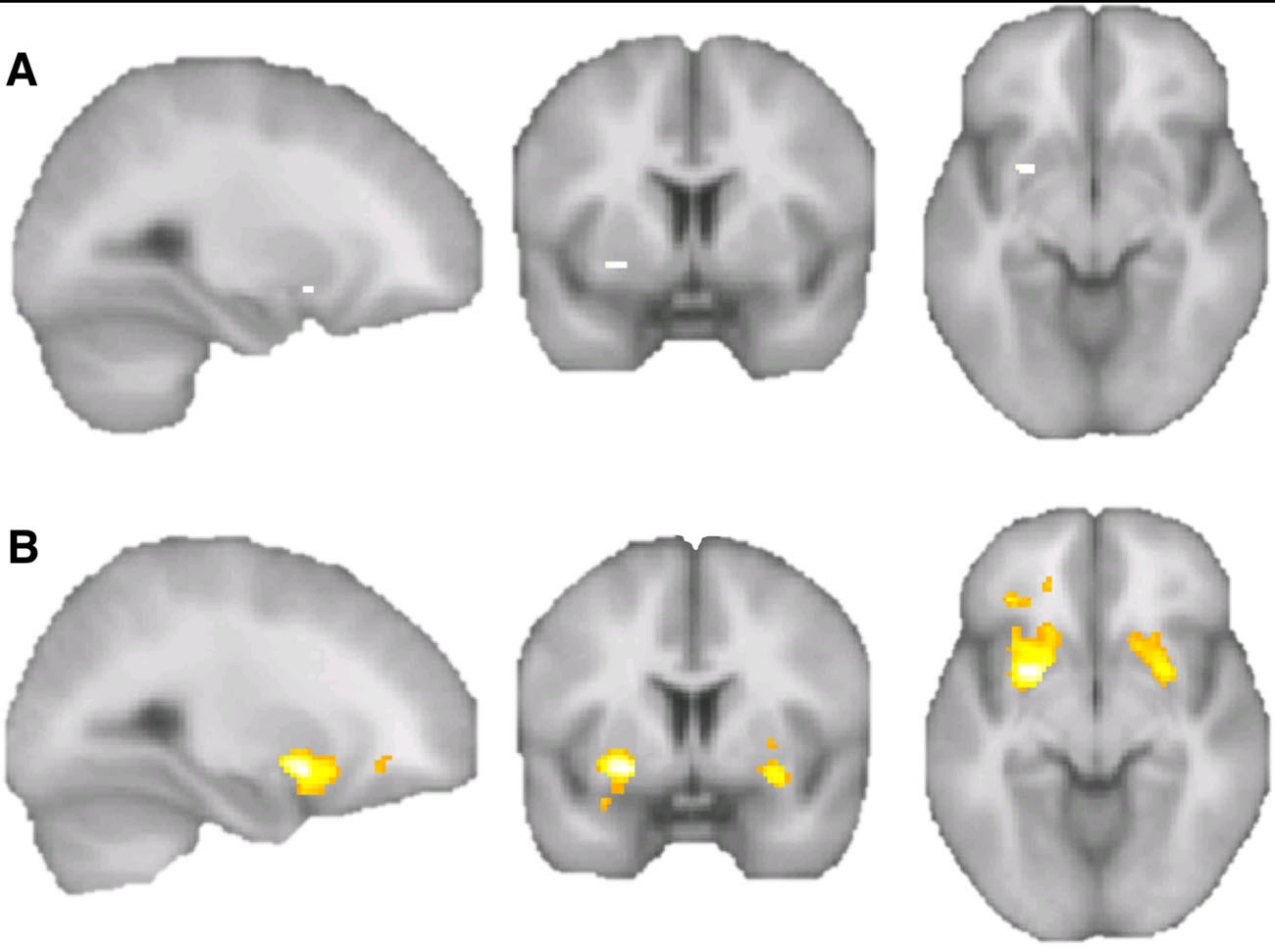
Whole-brain regression between summary scores of BMI, DA-D2R 141, and Taq1A genotype score with prediction error (PE) maps; no mask was applied. **a** Threshold *p* < 0.05 FWE corrected; one significant cluster, *x* = 26, *y* = 4, *z* = −8, peak *p*_FWE_ < 0.018, *k* = 8, right putamen, cluster *p*_FWE_ < 0.002. **b** An additional regression at lower threshold (*p* < 0.001, uncorrected) indicated that the BMI and genotype score correlated almost exclusively with ventral putamen PE activation (cluster *p*_FWE_ < 0.001 and *p* < 0.002, right and left hemispheres, see supplemental material for full results)

We further computed a summary score (addition, supplemental material) for the DA-D2R Taq1A alleles, −141C Ins/Del alleles, and BMI, and performed a whole-brain regression with PE regression maps. That showed a cluster in the right putamen (*p* < 0.05, FWE corrected, whole brain unmasked; Fig. 2, Supplemental Table 1), which was significant at the cluster level (*p* < 0.002). An additional exploratory analysis at lower significance threshold (*p* < 0.001 uncorrected, 50 voxel cluster threshold) indicated across the whole brain almost exclusive regression with putamen brain response, although there were three additional smaller clusters in the middle and inferior prefrontal cortex. In that analysis, the bilateral putamen clusters were significant *p* < 0.05 FWE corrected at the cluster level (right *p* < 0.001, left *p* < 0.002).

## Discussion

This study provides empirical evidence that DA-D2R alleles contribute together with BMI to human PE response in the putamen. The DA-D2R 141 Ins/Ins and Taq1A A2/A2 alleles and higher BMI were associated with lower PE response. DA-D1R allelic variation might be a factor in nucleus accumbens response, but we did not find evidence that DAT, COMT, or DA-D4R contribute to this response. Previous studies using computational algorithms found that in particular the putamen responds to PE tasks^4,5,28^. Our results are consistent with those findings.

From these results, we can propose a model for how the combination of genotype and BMI can explain a significant amount of variance in PE response. This is novel and has not been shown before. This is important for various reasons. First, it highlights the importance of DA-D2R allele frequency and proposing a mechanism for variation of PE response across individuals. Second, the importance of the DA-D2R in general PE response indicates that DA-D2R active medication may be useful in modifying PE response, which could have clinical implications. Third, the results suggest that variation in BMI is also associated with changes in PE, which is important as it suggests a mechanism for how eating behavior can affect DA-related brain response. Our study is a direct extension of the study by Eisenegger et al.^11^ that emphasized the DA-D2R and the Taq1A allele in reinforcement learning in humans^11^. That study investigated male participants only, while our study was restricted to adult females. Sex and also the state of menstrual cycle modify brain reward response, and future studies need to investigate those differences further across sexes^29^.

The NIMH RDoC project includes the PE model and this paradigm has been now applied in a host of studies including depression and psychotic disorders^30,31^. This makes it even more important to understand the factors that drive the PE signal, in order to properly contrast brain response between healthy individuals and those with specific psychiatric disorders. This study adds evidence to the long hypothesized genetic underpinnings, specifically DA-D2R alleles, driving PE response^9^. The DA-D2R adapts to food intake and can be modified by the type of diet and weight gain^16,32^. This has important implications not only for conditions such as obesity and eating disorders, but also for depression and schizophrenia, conditions also often associated with high or low weight^16,33^. It may therefore be key to assess DA receptor genotype and BMI, and take those effects into consideration when analyzing and interpreting studies that use this paradigm. Our previous data showed that extremes of under and overweight are associated with opposite PE response^12^. Here we studied healthy control individuals and we believe that being able to demonstrate those effects across the spectrum of normal weight suggests a strong effect of the variables studied. Nevertheless, future studies will need to study genotype and DA-D2R effects on PE response across the spectrum of psychiatric populations.

## Limitations

Our study did not measure a specific behavior response. However, choice and motivation have been associated with DA-D2R and PE response^34^. We only studied females and a comprehensive study is needed across both sexes. The sample is modest and requires replication, and in order to mitigate this, we used bootstrap procedures. The effects of the DA-D1R alleles did not survive multiple comparisons, however in a larger sample, their effects could have been significant. The correlation results are primarily driven by the major allele homozygotes and the heterozygotes. The number of minor allele homozygote individuals is small and their true mean value for PE response is difficult to assess. The data from this healthy control sample do not allow any inference on how genotype and BMI interact with psychiatric illness effects. Basic research has implicated the DA-D2R in reinforcement learning response in brain regions including striatum, pallidum, nucleus accumbens core, and habenula, but human fMRI is not necessarily well positioned to make such fine distinctions^35–38^. The calculated PE was modeled as absolute or strength of PE and regressed with the brain response reflected in the parameter estimates as in previous studies^12,19^. This approach does not separate by positive and negative activation, but has been selected because it is uncertain whether DA receptor function can be separated for positive and negative PE response^10^. We included a broad age range in the analysis for study of a larger population sample. In order to test for age effects, we ran an additional exploratory analysis, which also included age in the model. However, while BMI effects remained significant for right (*p* < 0.001) and left (*p* < 0.031) putamen, there were no significant effects for age for right (*p* < 0.495)- or left (*p* < 0.695)-sided putamen PE response. We therefore do not believe that the age range confounded the study results.

In summary, these data suggest that DA-D2R allelic variation contributes to PE response in healthy women together with BMI. This suggests that studies that apply the PE model should take those variables into account.

## Electronic supplementary material

Supplemental Material
