# Supplementary material for: Dopamine D2 −141C Ins/Del and Taq1A polymorphisms, body mass index, and prediction error brain response
Source: Transl Psychiatry. 2018 May 23;8:102. doi: 10.1038/s41398-018-0147-1 (PMC5966465; doi:10.1038/s41398-018-0147-1)
Supplement: Supplementary file 1 — Supplemental Material [file 41398_2018_147_MOESM1_ESM.docx]

**Supplemental Material**

**Results**

Genotype data were within the Hardy-Weinberg equilibrium.

Multiple Regression Analyses for the D2R alleles, BMI, and regions of interest.

**Summary Score Calculation**

We further computed a summary score (addition) for the DA-D2R Taq1A alleles, -141C Ins/Del alleles and BMI and performed a whole brain regression with PE regression maps. For the DA-D2R 141 the Del/Del genotype was assigned a value of 1, the Ins/Del 2 and Ins/Ins 3; for DA-D2R Taq1A a value of 1 was assigned to the A1/A1, a 2 to the A1/A2 and a 3 to the A2/A2 genotype. Those values were added to the raw BMI value and regressed with brain response.

Supplemental Table 1.

A. Results from regression analysis of DA-D2R genotype 141Ins/Del, Taq1A and BMI at threshold p<0.05 Peak FWE corrected.

B. Results from regression analysis of DA-D2R genotype 141Ins/Del, Taq1A and BMI at threshold p<0.001 uncorrected, 50 voxel cluster threshold.
